# Movement Performance is Associated With Dementia in Older Women: A 20 Year Longitudinal Study Using Optoelectronic Kinesiology

**DOI:** 10.1016/j.mcpdig.2026.100370

**Published:** 2026-05-20

**Authors:** Michael Matousek, Miia Kivipelto

**Affiliations:** Division of Clinical Geriatrics, Department of Neurobiology, Care Sciences and Society, Karolinska Institute, Stockholm, Sweden

## Abstract

**Objective:**

To use a motion capture technique in a longitudinal study design to study movement performance in older women and it’s association with incipient dementia.

**Patients and Methods:**

This study is part of the Gothenburg H70 birth cohort studies and the prospective population study of women in Gothenburg. Altogether 692 nondemented women aged 62-84 years were followed for 20 years. Movement performance was measured by the Posturo-Locomotion-Manual test using optoelectronic technology. Dementia diagnoses were based on information from neuropsychiatric examinations, informant interviews, hospital records, and registry data.

**Results:**

Total 170 women developed dementia during the follow-up. Slower gait at baseline (1992/1993) was associated with higher risks of dementia (1992-2012). Individuals in the second and third tertiles of the locomotor phase were associated with dementia.

**Conclusion:**

Our findings suggest that motor impairment is an important symptom of preclinical dementia in women.

Dementia is common in older people, and the prevalence is increasing, as the older population is increasing.^1^ It is important to identify persons at risk of dementia early in order to initiate early intervention, preferably before non-reversible cognitive symptoms appear.

The coexistence of cognitive complaints and slow gait is common, with a prevalence of 10% among older adults over 60 years without dementia.^2^ Deteriorated gait seems to appear early in the development of dementia and may precede the cognitive impairment.^3^^,^^4^ Discrete gait characteristics have been suggested to be early biomarkers to aid diagnostic algorithms in dementia.^5^

In this study, movement performance was measured by a postural-locomotion-manual test (PLM test) by optoelectronic technique. Previous studies have shown that the PLM test is a useful and valid tool to measure movement performance in patients with movement disorders, such as Parkinsons disease^6^, ^7^, ^8^ and other neurological disorders^9^^,^^10^ as well as in older people without movement disorders.^9^^,^^11^, ^12^, ^13^, ^14^ The test enables precisely simultaneous measurement of arm and leg movements and their mutual coordination in an everyday task. To the best of our knowledge, the objective PLM test has not been used earlier to study incipient dementia.

On the basis of a population-based cohort followed over 20 years, the study aimed to examine whether poor movement performance measured by PLM was associated with higher risks of incident dementia. We examined the association between movement performance at baseline in 1992 and incident dementia during follow-up 1992 to 2012.

## Patients And Methods

### Participants

The study is part of the H70 birth cohort studies in Gothenburg^15^^,^^16^ and the prospective population study of women in Gothenburg.^17^ The prospective population study of women is a longitudinal population study of women, which was initiated in 1968, and the H70 study is a longitudinal gerontological and geriatric population study of older people, which was initiated in 1971.

Altogether 1385 women aged 62-84 years were invited to participate in the population study in 1992/93 (ie, baseline investigation in this study).

The response rate was 66.5% (n=921) in the study as a whole, and 697 (50.3%) took part in the PLM test. Five women with dementia were excluded, leaving 692 women for the study (n=228 [62-year-olds], 233 [70-year-olds], 165 [74-year-olds], 55 [78-year-olds], and 11 [84-year-olds]). Follow-up examinations were conducted in the years 2000-2001, 2005, and 2009. Journal data from hospital admission were collected for the years 1992-2012, and all available data were finally combined to estimate incidence of and time to dementia. Furthermore, a subgroup of women (n=204; 122 [62-year-olds] and 82 [70-year-olds]) were examined with the PLM test both in 1992/93 (at ages 62 and 70 years) and 2000/01 (at ages 70 and 78 years). None of these women in the subgroup (n=204) were demented at both examinations. Nonparticipation in the PLM test was due to difficulties, eg, poor balance, to perform the test. Compared with the participants, the nonparticipants had a higher average age, more dependence in daily life activities, and more diseases, including hypertension, orthostatic hypotension, coronary heart disease, cerebrovascular disease, chronic bronchitis, cancer, depression, and arthritis.

### Diagnoses of Dementia

Diagnoses of dementia were based on combined information from neuropsychiatric examination, close informant interview, and hospital register.^18^^,^^19^ Neuropsychiatric examinations were performed by experienced psychiatrists in 1992/93. All examinations were semistructured. The examinations included ratings of common signs and symptoms of dementia, eg, assessments of memory, orientation, general knowledge, apraxia, visuospatial function, understanding proverbs, following commands, naming ability, and language. Identical instruments were used since 1992, including the comprehensive psychopathological rating scale,^20^ the mini mental state examination,^21^ the Alzheimer’s disease assessment scale,^22^ and the clinical dementia rating.^23^ Close informant interviews were performed by psychiatric nurses. The interviews were semistructured and comprised questions about changes in behavior and intellectual function, psychiatric symptoms, activities of daily living, and, when relevant, age at onset for stroke and dementia and disease course. The Swedish Hospital Discharge Register provided information on diagnoses of all individuals discharged from hospitals on a nationwide basis. Diagnoses were classified according to the International Statistical Classification of Diseases and Related Health Problems.

Dementia was diagnosed according to the Diagnostic and Statistical Manual of Mental Disorders criteria, as described previously.^18^^,^^19^ Dementia diagnoses were made by psychiatrists after reviewing information from both neuropsychiatric examinations and the close informant interview. The diagnosis was made if the participant had dementia according to both sources of information or if there was clear evidence of dementia from one source and subthreshold symptoms from the other. For individuals lost to follow-up, dementia diagnoses were based on information from the hospital discharge register. Age of dementia onset was based on the combined information from neuropsychiatric examination, close informants, and register data. All data from the period 1992-2012 were evaluated 2012, and the final dementia diagnoses were determined.

Subtypes of dementia were not diagnosed because of a small sample size. Stroke was defined as a self-reported history of stroke, stroke diagnosis in the hospital discharge register or infarcts seen on computed tomography. Diagnosis of stroke in our population studies has been described previously.^18^

### Confounding Variables

The information on medical conditions was obtained either from the subject’s self-report or from a medical examination performed by a physician. The definitions of diseases have been presented previously.^15^, ^16^, ^17^ Descriptive statistics of confounding variables are shown in Table 1. Coronary heart disease was defined as meeting one or more of the following criteria: angina pectoris according to a questionnaire, history of myocardial infarction, and electrocardiogram evidence of ischemia. Cerebrovascular diseases included a history of stroke, transient ischemic attacks, and brain infarcts on computed tomography. Hypertension was defined as a history of treatment with antihypertensive medicines or diastolic blood pressure >95 mm Hg measured at the baseline examination. Orthostatic hypotension was defined as a decline of ≥20 mm Hg in systolic blood pressure or ≥10 mm Hg in diastolic blood pressure from a supine position to an upright posture. Chronic bronchitis was assessed using a questionnaire. Arthritis was defined as a history of rheumatoid arthritis or osteoarthritis of the hip joint. Cancer was defined as any kind of cancer previously or presently. Depressive disorders were diagnosed according to Diagnostic and Statistical Manual of Mental Disorders.

**Table 1:** Descriptive Statistics of Confounding Variables

Variables	Outcome 1	Outcome 2
Age (y)	62-70	74-84
Education (%)		
Mandatory	62	69
Socio-economic status (%):	38	31
Low	42	42
Middle	49	45
High	9	13
Smoking (%):		
Never	54	73
Ex-smoker	26	16
Current	20	11
Coronary heart disease (%)	14	25
Cerebrovascular disease (%)	8	15
Hypertension (%)	29	34
Orthostatic hypotension (%)	11	20
Chronic bronchitis (%)	10	10
Arthritis (%)	14	16
Cancer (%)	12	12
Depressive disorder (%)	17 (only age 70)	15

### The Postural-Locomotion-Manual test

Movement performance in the PLM test (Figure) was measured by a noninvasive optoelectronic technique using infrared light (Qualisys Company, Sweden).

**Figure:**
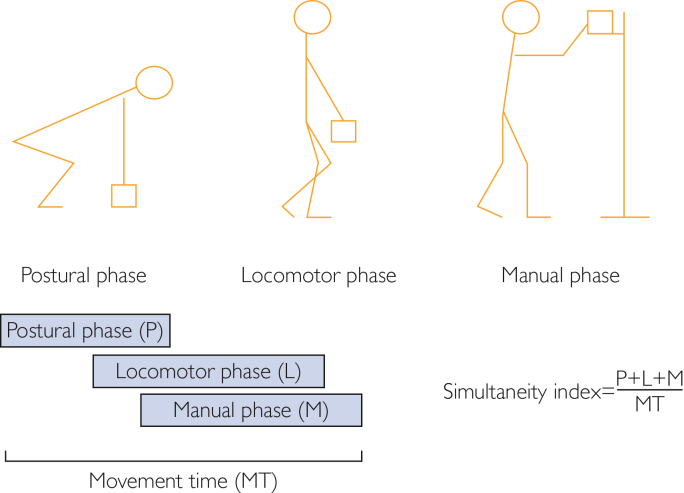
The Posturo-Locomotion-Manual test.

Reflective markers are placed on the right side of the head, shoulder, elbow, hip, ankle, and left foot. A seventh marker is placed on the test object; a metal handle fastened to a cylindrical horizontal plate weighing 550 g. A camera system registers the infrared light pulses reflected from the markers. The position of the markers was calculated 50 times/s as 2-dimensional (x, y) Cartesian room coordinates and was stored in a computer.

During the PLM test, the participants picked up the object from the floor, walked forward 1.5 meters and placed it on a shelf at the height of their chin. After that, they immediately went back to the starting-point carrying the object. They were asked to repeat this motor act continuously during a 30-second period. This was defined as one trial, and each subject had 3 trials. The first trial was performed at a normal speed, and the subsequent two trials were done as quickly as possible. The trial with the shortest movement time (MT) was chosen as the subject’s final result. The time taken (1) to move the object from the floor to the shelf (MT), (2) to raise the body after the object is picked up (postural phase [P phase]), (3) to move the feet from start until stop in front of the stand (locomotor phase [L phase]), and (4) for the goal-directed active arm movement to lift up and place the object on the stand (manual phase [M phase]) were calculated. The overlap of the different phases is illustrated by using the simultaneity index (SI), as calculated from the sum of the P, L, and M phase durations divided by the MT. The SI is an indicator of coordination of different phases. The PLM test have been described in early studies.^7^^,^^12^

### Statistical Analyses

Factors of dementia incidence during a 20-year follow-up period are evaluated by the Cox Proportional Hazard Model, and results are presented with HRs and 95% CIs. Change between 1992 and 2000 in PLM for nondemented 1992 was tested with a paired t-test, and linear regression models were used to test if mean PLM change differed between incidence and non-incidence cases. Statistical software use was primarily Stata, version 14.

In multivariate analysis was adjusted for several relevant cofounding factors; age, education, socio-economic group, former and current smokers, and diseases including hypertension, orthostatic hypotension, coronary heart disease, myocardial infarct, cerebrovascular disease, chronic bronchitis, cancer, depression, and arthritis. As a sensitivity control, models were calculated after excluding individuals with early death and early dementia incidence, ie, less than 2 years after their investigation. This procedure did not change the main results.

## Results

Among 692 women without dementia examined in 1992-1993, 170 developed dementia during 8896 person-years of follow-up from 1992 to 2012. Diagnoses were made in 135 from clinical examination or close informant interview and in 35 from hospital register or medical records. The mean age of dementia onset was 83 years. The mean number of years from the baseline examination to dementia onset (all types of dementia) was 10.3 years (SD 4.8). The median risk time was for ages 62, 70, 74, 78, 84 and all; 19.5, 15.5, 11.5, 9.0, 5.5, and for all 16.0 years. The interquartile range of the follow-up duration for ages 62, 70, 74, 78, 84, and all was 17.5-20.0, 10.5-19.5, 8.0-16.5, 4.5-14.5, 2.5-9.5, and for all, 10.5-19.5 years. Baseline characteristics of the study population in 1992-93 are given in Table 2. Table 3 shows mean PLM values in relation to development of dementia

**Table 2:** Baseline Characteristics of Study Population (n=692) in Years 1992/93

Variables	Outcome
Age (y); mean ± SD	70 ± 5.7
PLM (mean ± SD, s)	
MT	2.35 ± 0.73
P	0.93 ± 0.18
L	1.65 ± 0.39
M	1.50 ± 0.44
SI	1.77 ± 0.16
Education (%):	
Mandatory/more than mandatory	35/65
Marital status (%):	
Married/unmarried or widowed	
	54/46
Social-economic status (%):	
Low	42
Medium	48
High	11
Smoking (%):	
Ex-smoker/current smoker	23/17
Alcohol consumption (%):	
Never or less than once/wk	51
Once or more than once a wk	49
Physical activity (%):	
Less than 4 h/wk	19
More than 4 h/wk	81
BMI (mean ± SD)	26 ± 4.2
Cholesterol (mean ± SD)	6.4 ± 1.1
Hypertension (%)	46
Diabetes mellitus (%)	4.6
Myocardial infarct (%)	4.1
COPD (%)	12

Abbreviations: COPD, chronic obstructive pulmonary disease; MT, movement time; P, postural phase; L, locomotor phase; M, manual phase; SI, simultaneity index.

**Table 3:** The PLM Results at Baseline in Groups of Nondemented (n=522) and all Demented (n=170)^a^^,^^b^

	MT	P	L	M	SI
Nondemented	2.31 ± 0.65	0.92 ± 0.18	1.63 ± 0.39	1.47 ± 0.39	1.77 ± 0.16
All-cause dementia	2.49 ± 0.92	0.95 ± 0.17	1.73 ± 0.39	1.57 ± 0.54	1.76 ± 0.17

a Movement time and phases are shown in seconds and standard deviations.

b Abbreviations: MT, movement time; P, postural phase; L, locomotor phase; M, manual phase; SI, simultaneity index.

### PLM Versus Mini Mental State Examination

The 62-year cohort comprised approximately one third of the sample, and cognitive testing were not performed in this age group in 1992. In 448 individuals (at ages 70, 74, 78, and 84 years) with Mini mental state examination (MMSE) at baseline 1992/93, 401 had MMSE total score between 27 and 30 and 47 between 23 and 26. Linear regression model was calculated at baseline adjusted for age, and MMSE (total score range: 23-30) was dependent variable. Significant correlations were found between MMSE total score and MT (*P*<.05) as well as SI (*P*<.01).

### PLM Versus Dementia

Table 4 shows tertiles of the PLM in 1992 in relation to dementia between 1992 and 2012. Individuals in the second (hazard ratio [HR] 1.80; *P*=.006) and third tertiles (HR1.61; *P*=.028) of the L phase (ie, gait) had an increased risk for all-cause dementia when compared with those in the first tertile.

**Table 4:** Association Between Dementia (n=170) by 2012 and the PLM Test at Baseline (n=648) in 1992/93^a^^,^^b^

PLM variables	First tertile	Second tertile	Third tertile
Total dementia			
No. of cases	170		
Movement time	1	1.15 (0.76-1.75)	1.31 (0.86-2.0)
Postural phase	1	1.18 (0.80-1.75)	1.18 (0.80-1.73)
Locomotor phase	1	1.80 (1.19-2.72)∗∗	1.61 (1.05-2.47)∗
Manual phase	1	1.05 (0.71-1.57)	1.18 (0.79-1.76)
Simultaneity index	1	1.0 (0.69-1.47)	1.19 (0.81-1.73)

a HRs (HR, 95% CIs).

b Adjusted for age, education, socio-economic group, former or current smokers, and anamnesis of myocardial infarct.

∗ *P*<.05.

∗∗ *P*<.01.

The relation between the L phase (ie, gait) in the PLM test and risk of dementia was not linear. An association was found when comparing tertiles of the L phase. In the first tertile of L phase the risk of dementia was considerably lower, closed to halved, when compared with the higher tertiles, showing only small differences between tertiles 2 and 3.

### Association Between PLM and MMSE to Dementia Diagnoses

The results at baseline 1992/93 in 421 individuals with both MMSE and PLM showed that the L phase in tertiles 2 and 3 compared with tertile 1 was associated to incident dementia in the period 1992-2012 (HR, 1.97; *P*=.003), whereas the MMSE total score using tertile analysis was not associated to incident dementia in the same period. However, those with MMSE<26 showed doubled risk (HR=2.1; *P*=.002), but they compromised only 10% of the sample of nondemented at baseline.

Furthermore, in 626 individuals at baseline in 1992/93 the L phase in tertile 2 (HR, 1.70; *P*=.015) and tertile 3 (HR, 1.64; *P*=.031) was associated with subsequent occurrence of stroke (ie, 157 events) in the period 1992-2012.

### Change in PLM From 1992 to 2000 in Relation to Incident Dementia From 2002 to 2012

Altogether 204 women were free from dementia both in 1992/93 and in 2000/01 and were investigated with the PLM test on both occasions. Among those, 32 developed dementia during 2342 person-years of follow-up from 2002 to 2012. The mean age of dementia onset was 82.5 years. The mean number of years from the baseline examination to dementia onset was 14.1 years (SD 2.5).

The relation between change in PLM 1992-2001 and incidence dementia 2002-2012 is shown in Table 5. There was a general increase in PLM measures from 1992 to 2001, but those who developed dementia increased more in the MT (*P*<.01) and M phase (*P*<.01) than those who did not develop dementia. The sample size was too small to analyze subtypes of dementia separately.

**Table 5:** The subjects (n=204) investigated by the PLM test both 1992/93 and 2000/01

PLM variables	Nondemented (n=172)^a^^,^^c^		Delta (sd)	Subsequently demented (n=32)^a^^,^^c^		Delta (sd)	Difference in changes in both groups^b^^,^^d^
	1992/93	2000/01		1992/93	2000/01		
MT	2.06 ± 0.37	2.13 ± 0.42∗	0.068 ± 0.031	2.13 ± 0.38	2.43 ± 0.47∗∗	0.30 ± 0.096	*P*=.005∗∗
P	0.89 ± 0.16	0.90 ± 0.14 ns	0.015 ± 0.013	0.91 ± 0.11	0.96 ± 0.14 ns	0.050 ± 0.028	*P*=.27
L	1.48 ± 0.27	1.57 ± 0.28∗∗∗	0.085 ± 0.020	1.55 ± 0.26	1.74 ± 0.37∗	0.18 ± 0.073	*P*=.09
M	1.33 ± 0.28	1.37 ± 0.27ns	0.041 ± 0.026	1.35 ± 0.30	1.57 ± 0.28∗∗	0.21 ± 0.064	*P*=.009∗∗
SI	1.82 ± 0.14	1.83 ± 0.15 ns	0.010 ± 0.013	1.81 ± 0.13	1.77 ± 0.13 ns	0.032 ± 0.029	*P*=.19

a PLM results in nondemented (n=172) and subsequently demented (n=32) the period 2002-2012. Difference in changes in the PLM test in nondemented and subsequently demented between 1992/93 and 2000/01. Movement time and phases are shown in seconds and SDs.

b Abbreviations: MT, movement time; P, postural phase; L, locomotor phase; M, manual phase; SI, simultaneity index; MMSE, mini mental state examination; ns= not significant.

c Adjusted for age.

d Linear regression model adjusted for age, education, socio-economic group, former or current smokers and anamnesis of myocardial infarct.

∗ *P*<.05.

∗∗ *P*<.01.

∗∗∗ *P*<.001.

## Discussion

In a 20-year longitudinal population study, we found that older women who later developed dementia and stroke had slower movement at baseline. Furthermore, the motor speed in the upper extremities decreased more than in the lower extremities before the onset of dementia when compared with those who did not develop dementia. The relation between the L phase (ie, gait) in the PLM test and risk of dementia was not linear. An association was found when comparing tertiles of the L phase. In the first tertile of the L phase the risk of dementia was considerably lower, close to halved, when compared with the higher tertiles, showing only small differences between tertile 2 and 3. The data implicates that gait starts to impair early in the development of dementia. There was a relation between MT in the PLM test and MMSE at the baseline. Of interest is that the SI also was associated to MMSE, as it has earlier been found to measure parkinsonism.^6^^,^^10^ However, the following change in motor performance was not accompanied by a corresponding change in global cognitive function represented by the MMSE total score. Interestingly, the discreet difference in movement performance was seen more than 10 years prior to dementia diagnoses. Our findings suggest that motor performance constitutes important symptoms of preclinical dementia.

Several other studies have shown relations between motor impairment and cognitive decline in older adults. One cross-sectional study^24^ showed that individuals with mild cognitive impairment (MCI) performed worse on tasks involving fine and complex motor function (eg, tracking and manual dexterity) than individuals with normal cognitive function. Individuals with cognitive complaints, slow gait, preserved activities of daily living, and absence of dementia had an increased risk of developing vascular dementia.^5^ A longitudinal study^25^ evaluated motor performance prospectively for 3 years in a group of old people with normal cognitive function. Those who developed cognitive impairment during follow-up had slower finger tapping and took a longer time to walk 30 feet at baseline than those who remain cognitively intact. Another longitudinal study^26^ compared the trajectory of motor decline, as measured by gait speed and finger tapping speed, between old people who developed MCI and those who remained cognitively intact. Decline in gait speed accelerated 12 years before the onset of MCI, whereas decrease in finger tapping speed was seen after the onset of MCI. Gait speed decline has been found to precede cognitive decline, is linked to Alzheimer pathology, and might be used for early detection of an increased risk for dementia development.^3^^,^^4^ Further support for the claim that motor function is affected in preclinical dementia comes from a report^27^ that slower gait speed is associated with regional Aβ deposition in the frontal, striatal, temporal, parietal, anterior cingulate, precuneus, and occipital cortices among older people without dementia.

There are several possible explanations why poor baseline PLM performance was related to dementia. First, PLM test is a movement test but also a complex cognitive test. Performance of the test requires an interplay of multiple cognitive domains, such as attention, executive function, visuospatial, and motor processing function. Impairment of these cognitive domains may thus lead to poor performance in the PLM test. A similar association between the PLM test and cognitive tests was found in another study.^14^ Second, our earlier cross-sectional studies in women without dementia found that a slower L phase was related to the severity of white matter lesions and the M phase was related to temporal lobe atrophy,^12^^,^^13^ suggesting that PLM performance is related to both cerebral vascular damage and neurodegenerative changes. This supports our finding that change in the M phase during follow-up was associated with the incidence of dementia. Third, PLM tests might also be a general index of cardiovascular fitness, which has previously been related to the risk of dementia.^28^ Fourth, our findings may also be due to changes in visuo-motor function. Decline in visual processing capacity has been shown in MCI and Alzheimer’s disease,^29^ visual evaluation deficits measured with an infrared eye tracking test was associated to early dementia in another study,^30^ and individuals with preclinical dementia took a longer time to complete the visuomotor coordination task (visually guided goal-directed movement) than cognitively normal controls.^31^ Furthermore, the poor performance in visually guided goal-directed movement were related to a low level of cerebrospinal fluid amyloid β-protein 42 (AB42) among cognitively normal individuals.^31^

Among the strengths of this study are the representative sample, the long follow-up, the use of the PLM test, which enables detection of subtle movement impairment, 2 measurements of movement performance, which made it possible to examine change before clinical dementia, and the comprehensive examinations to diagnose dementia during follow-up. There are also some limitations. First, the sample size was small and it was not meaningful to assess subgroups of dementia in the longitudinal study of PLM. Second, the population included only female participants, and the findings cannot be generalized to male populations. Third, response rates at baseline were 66.5%. It is possible that our sample was healthier than the general female population.

Further use of the PLM test might be to distinguish the Alzheimer’s disease and Lewy body dementia at early stages, as it might detect inconspicuous signs of parkinsonism not obvious to the naked eye in patients developing Lewy body dementia. The knowledge obtained by optoelectronic kinesiology might be used in screening for dementia by general practitioners. Repeated annual walking tests combined with tests of upper extremities, eg, finger tapping tests, might give valuable information for incipient dementia swiftly and at low cost.

## Conclusion

We found in a 20-year longitudinal population study of female participants that a subgroup of older women had slower motor speed at baseline and an increased risk to develop dementia. Moreover, deterioration of function in upper extremities related to subsequent dementia. However, the population included only female participants, and thus the findings cannot be generalized to male populations. Our findings suggest that motor impairment is an important symptom of preclinical dementia. It is of interest to further study whether diverse discreet changes of movement performance in older people precede cognitive symptoms in the development of various subtypes of dementia.

## Potential Competing Interests

Dr Matousek declares no conflicts of interest. Dr Kivipelto reports research support from Academy of Finland, Swedish Research Council, Joint Program of Neurodegenerative Disorders, IMI, Knut and Alice Wallenberg Foundation, Center for Innovative Medicine, Stiftelsen Stockholms Sjukhem, Konung Gustaf V:s och Drottning Victorias Frimurarestiftelse, Alzheimer’s Research and Prevention Foundation, Alzheimerfonden, Hjärnfonden, Region Stockholm, and Leif Lundblad Foundation grants. Dr Kivipelto is part of a guidelines development group in WHO, a Governance Committee member of the Global Council on Brain Health, and is on the advisory board for Combinostics, Roche, and Biogen.

## Ethics Statement

The Ethics Committee of the University of Gothenburg approved the study. All participants gave informed consent to participate, in accordance with the provisions of the Helsinki Declaration.
